# A Computational Model of Similarity Analysis in Quality of Life Research: An Example of Studies in Poland

**DOI:** 10.3390/life12010056

**Published:** 2022-01-01

**Authors:** Agnieszka Bielińska, Piotr Wa̧ż, Dorota Bielińska-Wa̧ż

**Affiliations:** 1 Department of Quality of Life Research, Medical University of Gdańsk, 80-210 Gdańsk, Poland; agnieszka.bielinska1984@gmail.com; 2 Department of Nuclear Medicine, Medical University of Gdańsk, 80-210 Gdańsk, Poland; phwaz@gumed.edu.pl; 3 Department of Radiological Informatics and Statistics, Medical University of Gdańsk, 80-210 Gdańsk, Poland

**Keywords:** health informatics, data analysis, clustering, correspondence analysis, quality of life, World Health Organization Quality of Life-BREF

## Abstract

Due to the multidimensional structure of the results of similarity studies, their analysis is often difficult. Therefore, a compact and transparent presentation of these results is essential. The purpose of the present study is to propose a graphical representation of the results of similarity analysis in studies on the quality of life. The results are visualized on specific diagrams (maps), where a large amount of information is presented in a compact form. New similarity maps obtained using a computational method, correspondence analysis, are shown as a convenient tool for comparative studies on the quality of life of different groups of individuals. The usefulness of this approach to the description of changes of the quality of life after the retirement threshold in different domains is demonstrated. The World Health Organization Quality of Life-BREF questionnaire was used to evaluate individuals in Poland. By analyzing clusters on the similarity maps, two groups (employees and retirees) were classified according to their quality of life in different domains. By comparing the structures of the classification maps containing the information about the whole system considered, it is clearly seen which factors are important in the comparative studies. For the considered problems, the uncertainty coefficients describing the effect size and preserving the information on the symmetry of the variables that were used for the creation of the contingency tables were evaluated.

## 1. Introduction

Research on the quality of life has grown in importance in recent years, along with a holistic and interdisciplinary view of the situation of individuals in both social and medical sciences. The main goal of research on the quality of life is to understand the subjective feelings of a person, as well as to determine what factors have a decisive influence on him/her. The quality of life is a multidimensional and interdisciplinary concept, so an adequate definition and evaluation have been elusive. There are many definitions and different methods to measure this hard-to-grasp value. The issue of the quality of life was discussed in antiquity. Ancient philosophers wondered on what a good and happy life depends.

The beginning of research on the quality of life in the modern sense dates back to the 1960s, when this concept appeared in social and economic sciences. In medicine, the increased interest in the quality of life of patients took place in the second half of the 20th Century. Oncology patients were initially the focus of attention due to the significant impairment of daily functioning caused by both the disease and the therapy itself. In the late 1940s, David Karnofsky studied the well-being and the possibilities of self-care of cancer patients [1]. In 1960, Charles Zubrod et al. focused on functional disorders of cancer patients, based on their performance status [2]. In the 1970s, the concept of the quality of life appeared in medical nomenclature. In 1975, it became a keyword in the Medline—medical bibliographic database. Two years later, the term “quality of life” was included in Index Medicus. The definition of the health-related quality of life was introduced into medical science by Harvey Schipper in 1990 [3]. He defined it as the impact of the disease and treatment on functioning and life satisfaction, from the patient’s point of view. According to this author, the quality of life is determined by factors such as: the patient’s physical condition and mobility, economic conditions, and somatic sensations, such as pain experienced during the disease.

It was in the 1990s when research on the quality of life flourished. The International Society for Quality of Life Research was established in 1993, with over 1200 members from over 40 countries, wherein 50% members reside in North America, 35% reside in Europe, and the remaining 15% of the members live around the globe [4]. The aim of the organization is to support research on health-related quality of life, improve the quality of health care, and promote a healthy lifestyle.

The studies on the quality of life have developed continuously, and many articles can be found in the literature. Let us mention several examples from recent years.

Dilek Baday-Keskin and Bilge Ekinci focused on the relationship between kinesiophobia and health-related quality of life in patients with rheumatoid arthritis [5]. Subhabrata Moitra et al. studied the influence of the physical environment in health-related quality of life in chronic obstructive pulmonary disease [6]. Tingting Gao et al. studied the relationship between childhood maltreatment and the quality of life [7]. Shady Abdelsalam et al. studied the association between depression and oral-health-related quality of life in people who inject drugs [8]. Leigh Tooths et al. investigated family contextual effects on the association among screen time, behavior, and health-related quality of life in child siblings [9]. Andres Redondo-Tebar et al. analyzed the differences in health-related quality of life between typically developing children and children with developmental coordination disorder. In the studies, parents’ and children’s perceptions of the quality of life were considered and compared [10]. Ryan Haggart et al. compared the health-related quality of life outcomes in gay and bisexual men following prostate cancer [11].

From a formal point of view, deriving information about the quality of life is a comparative (classification) analysis of the results depending on various factors for different groups of individuals. A graphical representation of the results applied in this work for the groups: retirees and employees, is general and may be a convenient tool for studies on the quality of life and its dependence on different factors for arbitrary groups of individuals.

Retirement is one of the most important critical moments related to changing one’s lifestyle. Recently, many articles presenting changes in different aspects of life that appear after the retirement threshold have been published. In particular, changes in physical activity related to the transition to retirement have been carefully studied [12,13,14,15]. Generally, some specific changes in health-related quality of life have been observed [16,17,18,19]. The correlation of health changes with age is natural. Retirement may influence this natural process and cause more rapid changes for different reasons, often strictly related to nationality. For example, Kail studied the impact of private insurance coverage on the symptoms of depression [18]. The author indicated that being without employment-based insurance is particularly problematic during the transition to retirement, implying some negative health consequences.

Retirement may have a negative impact not only on the health condition, but also on the quality of life. The World Health Organization (WHO) defines the quality of life as an individual’s perception of his/her position in life in the context of the culture and value systems in which he/she lives and in relation to his/her goals, expectations, standards, and concerns [20]. This definition emphasizes that the quality of life is a subjective evaluation of the individual’s life, which depends on the social, environmental, and cultural context. According to the WHO, the quality of life is affected by the person’s physical health, psychological state, personal beliefs, social relationships, and environment. Some studies have shown associations between retirement and changes in the quality of life in different domains [21,22,23,24]. Since job position is an important element of personal identity construction and the professional domain is important for self-description, retirement constitutes a major change in perceiving one’s self-image [25].

Are these changes always negative? Smeaton et al. asked: “Does Retirement Offer a ‘Window of Opportunity’ for Lifestyle Change?” [26]. The authors presented a British view. In the present article, we present a study of this problem from the Polish perspective.

The influence of the University of the Third Age (U3A) on different aspects of life in many countries has already been discussed by several authors, as for example [27,28,29,30,31]. In our recent studies on changes of the quality of life related to the transition to retirement, we also considered the intellectual activity of retirees (as, e.g., the attendance at lectures at the U3A) [32]. In these studies, we used two our own questionnaires: the Questionnaire for an Employed or a Self-Employed Person and the Questionnaire for a Retiree [32]. We also performed some pilot studies using the World Health Organization Quality of Life-BREF (WHOQOL-BREF) questionnaire [33,34,35,36]. In particular, we studied different factors influencing the quality of life such as gender [33] and marital status [34] in four domains included in the WHOQOL-BREF questionnaire. We also considered job position in the physical health and psychological domains [35]. This factor, in the social relationships and environment domains, has also been studied [36]. In our approach, groups of individuals and their answers to questions are considered as objects represented by points in properly constructed classification maps.

The problem of classification is related to similarity/dissimilarity analysis. This kind of interdisciplinary analysis allows us to derive information about the relations among different kinds of objects. In particular, we formulated such an approach by introducing some new mathematical definitions of the coordinate axes in the similarity maps for the studies of the properties of biological sequence [37], of stellar spectra [38], and of molecular spectra [39]. We used several kinds of classifiers—among others, the moments of different kinds of distributions, in particular the asymmetry coefficients [40].

In the present work, we continue the studies on the influence of retirement on the quality of life. The objects under consideration were groups of individuals and their answers to questions (about their quality of life) included in the WHOQOL-BREF questionnaire. Some new classification maps are shown in Section 3. It was demonstrated that a graphical representation of the results is a convenient tool for comparative studies of the quality of life of different groups of individuals.

## 2. Materials and Methods

The studied group consisted of 480 individuals, selected from Bydgoszcz, a city in Poland. This is the eighth largest city in Poland (about three hundred fifty thousand citizens). Each respondent was asked if she/he was a retiree or an employee. The retirees answered a question about the attendance at lectures at the U3A. The questionnaires of the individuals older than 50 were considered. Finally, we considered 449 individuals, including 160 employees (100 females and 60 males) and 289 retirees (186 females and 103 males).

All the participants were evaluated using the WHOQOL-BREF questionnaire, world-wide known as a standard tool in quality of life research [41,42,43,44,45,46]. The information from the participants used in the study was anonymous and was obtained in written form. The results were generated using the R statistics language [47,48]. The assumed significance level was α=0.05.

For the graphical representation of the results, we applied Correspondence Analysis (CA), a practical tool for processing the data set of our problem [49]. In this method, one creates maps in which the objects under consideration cluster in a specific way. The structure and the distribution of the clusters carry some information about the objects.

The starting point in CA is to record the observed number of counts of the categorical variables, in the form of a contingency table, which displays the frequency distribution of these variables:

(1) $$M=[f_{ij}],$$

where i=(1,2,…,r), j=(1,2…,c). Next, the following vectors were evaluated:

(2) $$v_{r}=\left[q_{j}\right],\;\;\;q_{j}=\sum_{j=1}^{c}\frac{f_{ij}}{f},$$

(3) $$v_{c}=\left[q_{i}\right],\;\;\;q_{i}=\sum_{i=1}^{r}\frac{f_{ij}}{f},$$

where *f* is the total number of counts. In order to indicate the coordinates that allow locating the points corresponding to the categorical variables in the space of a specific dimension, one should use singular-value decomposition. The decomposition proceeds as follows:

(4) $$\Lambda =\Delta_{r}^{-1/2}\left(Q-v_{r}v_{c}^{T}\right)\Delta_{c}^{-1/2}=\Pi \Gamma V^{T},$$

where $\Delta_{r}$ and $\Delta_{c}$ are diagonal matrices with elements $q_{j}$ and $q_{i}$, respectively. The matrix *Q* consists of elements $f_{ij}/f$. Γ is a diagonal matrix with non-zero elements, which are the singular values of matrix Λ. The matrices Π and *V* are left and right singular vectors of Λ. The non-zero singular values of the matrix Λ are the square roots of the non-zero eigenvalues of both $\Lambda \Lambda^{T}$ and $\Lambda^{T}\Lambda$. A space for the presentation of the relations between two categorical variables can be at most n-dimensional, n=min(r−1;c−1).

The coordinates of points representing the categorical variables recorded in the rows of the Θ matrix and in the columns of the Ξ matrix in the n-dimensional space were evaluated using the formulas:

(5) $$\Theta =\Delta_{r}^{-1/2}\Pi \Gamma$$

and:

(6) $$\Xi =\Delta_{c}^{-1/2}V\Gamma .$$

The rows of the Θ and Ξ matrices correspond to categorical variables recorded in the rows and columns of the contingency table *M*, respectively. The columns of the Θ and Ξ matrices correspond to the coordinates on the subsequent principal axes.

In the present work, the considered objects represented in the similarity maps were *groups* (*employees*, *retirees*) and *answers* (*positive*, *negative*, *neutral*) to questions included in the WHOQOL-BREF questionnaire. This questionnaire contains 26 questions— two questions are related to overall quality of life and general health and the remaining 24 questions, considered in this work and listed below, concern four domains: physical health, psychological, social relationships, and environment:

Physical health (Domain 1):

To what extent do you feel that physical pain prevents you from doing what you need to do?

How much do you need any medical treatment to function in your daily life?

Do you have enough energy for everyday life?

How well are you able to get around?

How satisfied are you with your sleep?

How satisfied are you with your ability to perform your daily living activities?

How satisfied are you with your capacity for work?

Psychological (Domain 2)

How much do you enjoy life?

To what extent do you feel your life to be meaningful?

How well are you able to concentrate?

Are you able to accept your bodily appearance?

How satisfied are you with yourself?

How often do you have negative feelings such as blue mood, despair, anxiety, depression?

Social relationships (Domain 3)

How satisfied are you with your personal relationships?

How satisfied are you with your sex life?

How satisfied are you with the support you get from your friends?

Environment (Domain 4)

How safe do you feel in your daily life?

How healthy is your physical environment?

Have you enough money to meet your needs?

How available to you is the information that you need in your daily-to-day life?

To what extent do you have the opportunity for leisure activities?

How satisfied are you with the condition of your living place?

How satisfied are you with your access to health services?

How satisfied are you with your transport?

One could choose one of five answers to each question: *A-1, A-2, A-3, A-4, A-5*. In the cases of Domain 3 and Domain 4, *A-1* corresponds to the least favorable quality of life and *A-5* the most favorable one. In some cases related to Domain 1 and Domain 2, the sequence of questions was different. Therefore, for convenience, we modified the notation so that the sequence of questions was consistent in all cases. Thus, in this report, symbols *V* correspond to Domains 1 and 2, while symbols *A* to Domains 3 and 4. Indices 1 and 2 refer to the *negative* answers, 3—to the *neutral*, 4 and 5—to the *positive*. Thus, for example, *V-4* stands for a *positive* answer in either Domain 1 or Domain 2.

The group of *retirees* was split into two subgroups: 183 non-students of the U3A (107 females and 76 males)*—retirees1*, and 106 students of the U3A (79 females and 27 males)*—retirees2*.

Finally, the objects represented as points on the classification maps considered in this work were: *employees*, *retirees1*, *retirees2*, and *V-i, A-i*, i=1,2…,5.

## 3. Results and Discussion

**Figure 1:**
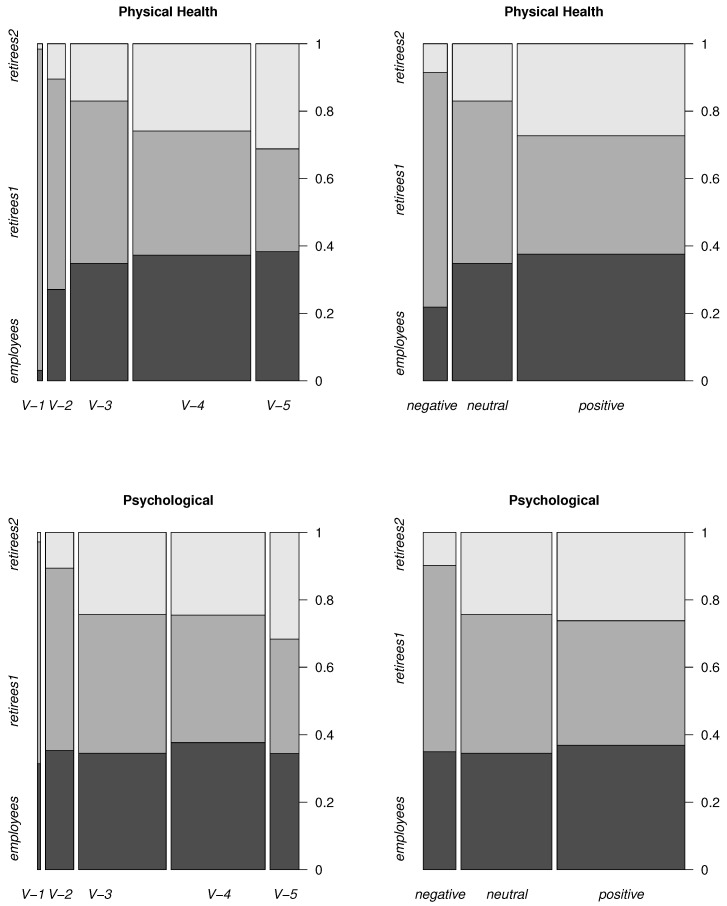
Number of particular answers for different groups (Domain 1, Domain 2).

**Figure 2:**
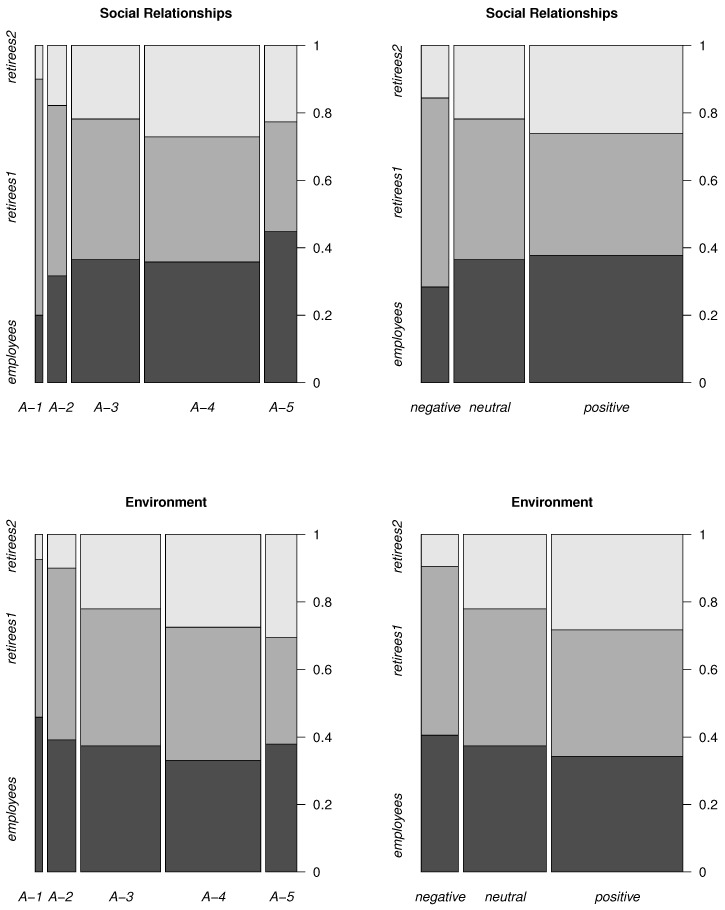
Number of particular answers for different groups (Domain 3, Domain 4).

Figure 1 and Figure 2 show the spine plots. Figure 1 describes the number of answers (normalized to one) of the respondents related to Domain 1 (top panels) and to Domain 2 (bottom panels). Similarly, in Figure 2 are represented Domain 3 (top panels) and Domain 4 (bottom panels). In the right plots, both negative answers (differing by the degree of negativeness) are combined into a single *negative*, and similarly, two positive answers are combined into one *positive*. The *neutral* one is equal to *V-3* (Domains 1 and 2) and to *A-3* (Domains 3 and 4).

The widths in the plots represent the total number of answers for all groups. As one can see, the width of the *positives* is the largest and that of the *negatives* is the smallest for all domains. This means that the total number of *positive* answers is the largest and the number of the *negative* answers is the smallest in all domains.

In the group of *employees*, the largest number of answers (compared to the number of other answers in this group) is *positive* in the domains physical health, psychological, social relationships and *negative* in the domain environment.

Considering *retirees1*, the largest number of answers (compared to the number of other answers in this group) is *negative* in all domains.

Considering *retirees2*, the largest number of answers (compared to the number of other answers in this group) is *positive* in all domains.

Table 1, Table 2, Table 3, Table 4, Table 5, Table 6, Table 7 and Table 8 show the input data for Figure 1 and Figure 2. Table 1 and Table 2 correspond to the physical health domain, Table 3 and Table 4 to the psychological domain, Table 5 and Table 6 to the social relationships domain, and Table 7 and Table 8 to the environment domain.

For the data presented in the tables and containing the information about the counts of particular answers (*A*, *V*, *negative, neutral, positive*) given by particular groups (*employees, retirees1, retirees2*) in different domains, the $\chi^{2}$ test of independence was performed. In all cases p<α.

The maximum value of the $\chi^{2}$ coefficient depends on the sample size and on the size of the contingency table. The coefficient can be normalized from 0–1. The normalized coefficient is independent of the sample size and of the size of the contingency table. For this purpose, several coefficients were defined. In this work, the uncertainty coefficient (Thiel’s U correlation coefficient) was chosen. This coefficient is normalized. Additionally, it preserves the information about the symmetry between the variables used for the creation of the contingency tables. The symmetric measures of the uncertainty coefficients *U* for each contingency table were calculated and are shown in the table captions. These coefficients appear to be similar for some specific pairs of the tables. For example, *U* in Table 1 is similar to *U* in Table 2; *U* in Table 3 is similar to *U* in Table 4, and so on. This means that the information remains similar after five answers (*V-i, A-i*, i=1,2…,5) are reduced to three (*negative, neutral, positive*).

The $\chi^{2}$ test of independence reveals only that there is a statistically significant association between variables. A more precise technique is correspondence analysis, designed to explore the relationships among categorical variables.

**Figure 3:**
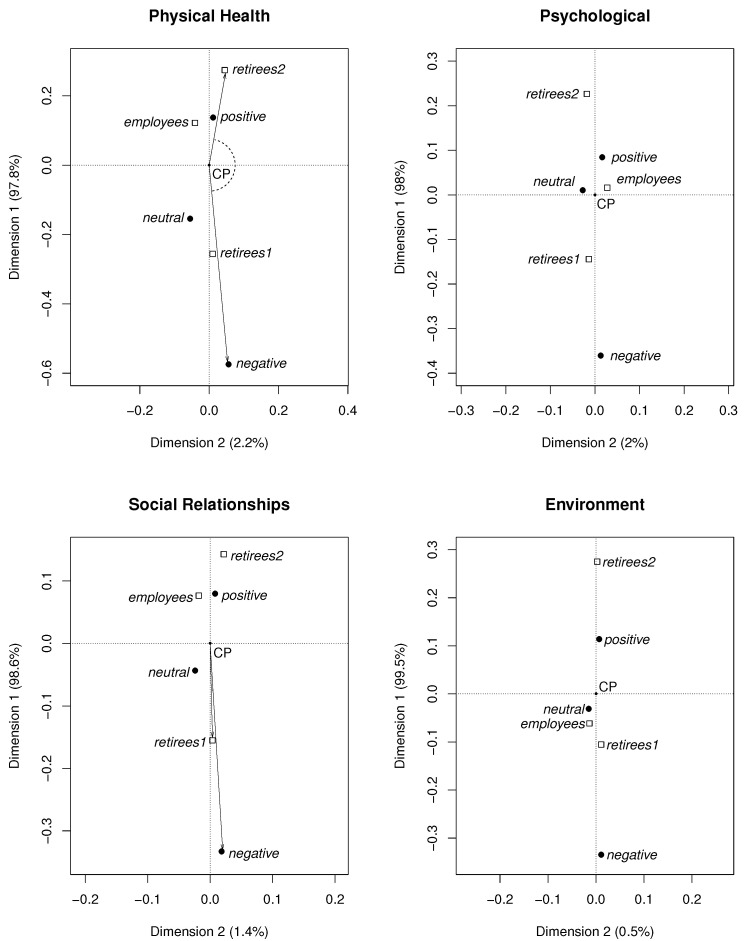
Maps obtained using the CA method (*answers*: *positive, neutral, negative*).

**Figure 4:**
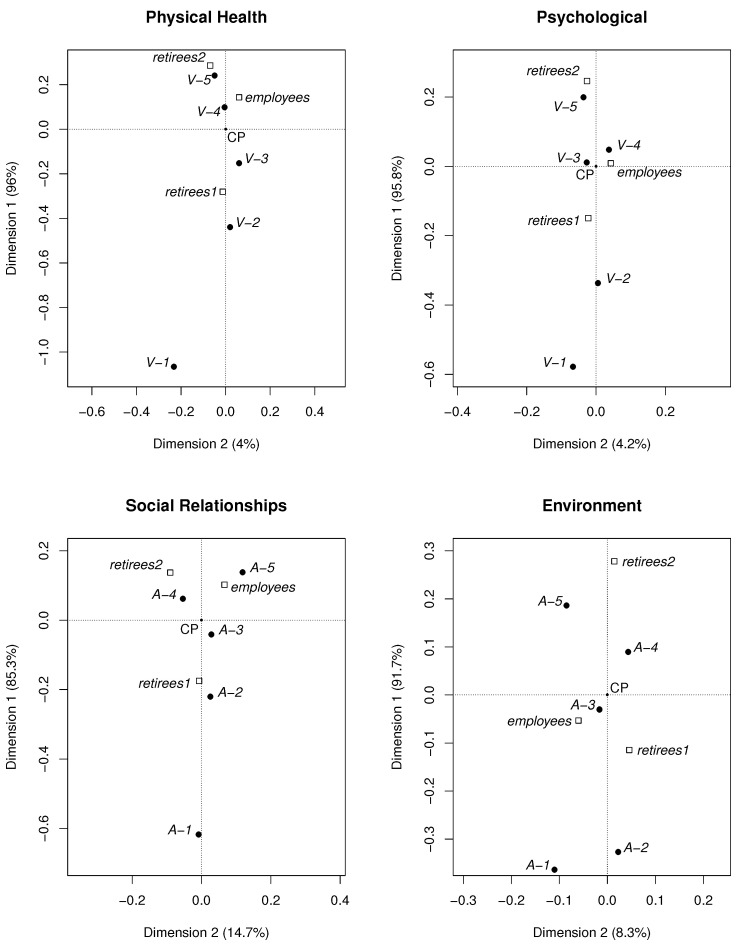
Maps obtained using the CA method (*answers*: *V-i, A-i*, i=1,2…,5).

Figure 3 and Figure 4 show the maps obtained using this method. Points in these maps represent particular groups and the answers. In the maps, the concentrations of points indicate associations among different *answers* (*A*, *V*, *negative, neutral, positive*) and particular *groups* (*employees, retirees1, retirees2*).

The concentrations of points in the maps are defined by the angles and the lengths of the appropriate vectors. The origins of all vectors are located at the central point denoted as CP, which is the crossing point of the dotted lines marked in the maps. The terminal points of the vectors represent the considered objects—empty squares (*groups*) and full circles (*answers*). A *group* and an *answer* create a cluster (positive association) if the angle between the vectors CP-square and CP-circle is small. Angles close to $90^{\circ }$ indicate no relationship. Angles close to $180^{\circ }$ indicate a negative association. The longer the vector, the stronger the corresponding (negative or positive) association is.

In Figure 3, examples of appropriate vectors corresponding to the negative association *retirees2—negative* (top left panel) and the vectors corresponding to the positive association, i.e., a cluster *retirees1—negative* (bottom left panel), are shown.

In Figure 3, the *negative, positive, and neutral* answers are displayed. Figure 4 shows *A-i* and *V-i*, i=1,2,3,4,5. In both figures, all considered groups, i.e., *employees*, *retirees1*, *retirees2*, are included.

The most frequent answers form clusters with the corresponding groups. As one can see, the clusters are:

- *retirees2—positive* (Domains 1, 2, 3, and 4);
- *retirees1—negative* (Domains 1, 2, 3, and 4);
- *employees—positive* (Domains 1, 2, and 3);
- *employees—neutral* (Domain 4).

The angles between vectors CP-*retirees2* and CP-*positive*, as well as between vectors CP-*retirees1* and CP-*negative* are small in all domains. Furthermore, the angles between CP-*employees* and CP-*positive*, as well as between CP-*employees* and CP-*neutral* are small, respectively, in Domains 1, 2, 3 and 4. Then, in each of theses cases, the corresponding points belong to the same cluster.

There are also negative associations (the angles between the vectors are close to $180^{\circ }$):

- *retirees2—negative* (Domains 1, 2, 3, and 4);
- *retirees1—positive* (Domains 1, 2, 3, and 4);
- *employees—negative* (Domains 1, 2, and 3);
- *employees—positive* (Domain 4).

The points representing *employees* are located closer to CP than the ones representing *retirees2* and *retirees1*. This means that the associations for the employees are weak in all clusters. The strongest (negative) associations are *retirees2—negative* in all the domains (the lengths of vectors CP-*retirees2* and CP-*negative* are the largest).

In Figure 3, the cumulative answers are shown (*positive, negative*), and in Figure 4, we can see some details. Finally, we obtained the following clusters (Figure 4):

- *retirees2—V-5* (Domains 1 and 2);
- *retirees2—A-4* (Domains 3 and 4);
- *retirees1—V-2* (Domains 1 and 2);
- *retirees1—A-1* (Domain 3);
- *retirees1—A-2* (Domain 4);
- *employees—A-5* (Domain 3).

The least common answers, i.e., the negative associations, for the corresponding groups, are as follows:

- *retirees2—V-2* (Domains 1 and 2);
- *retirees2—A-1* (Domain 3);
- *retirees2—A-2* (Domain 4).

If the points representing *employees* and *retirees* cluster with the same kind of answer, then the quality of life does not change after the retirement threshold. The points representing the employees are located close to the CP in all domains. This means that *employees* create only weak associations with *answers*. The attendance at lectures at the University of the Third Age is a very important classifier determining the quality of life for the retirees in all domains. Retirees attending the U3A evaluated their physical health as better than other retirees and also better than employees (stronger positive association *retirees2—positive* than *employees—positive*). The retirement threshold changes the quality of life of the retirees who do not study at the U3A.

It is interesting that also in the psychological domain, students of the U3A are more satisfied than the *employees*. Students of the U3A cluster with the most positive answer (*V-5*). In this case, we observed a positive influence of the retirement.

The slightly negative influence of the retirement was observed in the social relationships domain: the *employees* cluster with the most positive answer *A-5*, while the students of the U3A with *A-4* and non-students of the U3A with *A-1*.

Considering the environment, analogous to the other domains, students of the U3A estimated their quality of life as better than non-students.

All respondents who participated in the survey lived in Bydgoszcz—the eighth largest city in Poland. Thus, the study concerned only the inhabitants of a city—the inhabitants of villages and small towns were not represented in the research sample. The study was conducted among students of the University of the Third Age in Bydgoszcz, as well as among people who did not undertake this form of activity. Individuals from the second group were selected by the *snowball sampling* technique. The agreeable participants were asked to recommend other contacts who fit the research criteria and who potentially might also be willing participants. Individuals tend to recruit people they know well, and therefore, the respondents taking part in the study may have similar characteristics.

Though the computational model can be applied in comparative studies on the quality of life of arbitrary groups of individuals, the similarity maps can only be obtained if the dimensions of the contingency tables (Table 1, Table 2, Table 3, Table 4, Table 5, Table 6, Table 7 and Table 8) fulfill the following inequality:

(7) min(r−1;c−1)≥3,

where *r* is the number of rows and *c* is the number of columns.

## 4. Conclusions

In the present work, groups of individuals and their answers to questions were represented by points in a two-dimensional space. An abstract mathematical approach, CA, revealed new properties of the considered objects. In CA, the set of particular objects is treated as a single system. To obtain the maps, the information about all objects was taken into account simultaneously. Therefore, by studying the structure of the maps, we obtained a global description of the system. As a consequence, in a single map, a large amount of information may be stored.

In traditional approaches to studying correlations between different objects or processes, a substantial part of the available information may be lost. In the most common methods, only the linear correlation between variables is measured. If nonlinear correlations, as for example exponential or quadratic, exist in the system, the results may be entirely wrong. Therefore, using non-standard mathematical tools, capable of describing nonlinear effects, may lead to new, in some cases unexpected, results.

As already mentioned, classification maps have their analogs in the methods developed by us earlier in different areas of computational science, including bioinformatics characterization of SARS-CoV-2 [37]. By analyzing clusters in the similarity maps, we classified different kinds of objects, such as stars, chemical compounds (for example, persistent organic pollutants [39]), and biological sequences (for example, genome sequences of viruses). In the present work, groups of individuals were classified according to their quality of life in different domains. In particular, it is clearly seen in the classification maps that students of the U3A estimated their quality of life as better than non-students. This observation is a summary of our previous studies in which particular factors influencing these results were considered [33,34,35,36].

## Figures and Tables

**Table 1:** The number of answers in Domain 1 (Figure 1, top left panel; p<0.0001; U=0.027817).

	Employees	Retirees1	Retirees2	Total
*V-1*	2	61	1	64
*V-2*	62	143	24	229
*V-3*	254	352	124	730
*V-4*	557	551	387	1495
*V-5*	210	167	171	548
total	1085	1274	707	3066

**Table 2:** The number of answers in Domain 1 (Figure 1, top right panel; p<0.0001; U=0.027111).

	Employees	Retirees1	Retirees2	Total
*negative*	64	204	25	293
*neutral*	254	352	124	730
*positive*	767	718	558	2043
total	1085	1274	707	3066

**Table 3:** The number of answers in Domain 2 (Figure 1, bottom left panel; p<0.0001; U=0.011341).

	Employees	Retirees1	Retirees2	Total
*V-1*	11	23	1	35
*V-2*	110	168	33	311
*V-3*	333	396	235	964
*V-4*	390	391	254	1035
*V-5*	110	108	101	319
total	954	1086	624	2664

**Table 4:** The number of answers in Domain 2 (Figure 1, bottom right panel; p<0.0001; U=0.011331).

	Employees	Retirees1	Retirees2	Total
*negative*	121	191	34	346
*neutral*	333	396	235	964
*positive*	500	499	355	1354
total	954	1086	624	2664

**Table 5:** The number of answers in Domain 3 (Figure 2, top left panel; p=0.0001; U=0.010045).

	Employees	Retirees1	Retirees2	Total
*A-1*	8	28	4	40
*A-2*	32	51	18	101
*A-3*	132	151	79	362
*A-4*	219	227	166	612
*A-5*	77	56	39	172
total	468	513	306	1287

**Table 6:** The number of answers in Domain 3 (Figure 2, top right panel; p=0.0002; U=0.008407).

	Employees	Retirees1	Retirees2	Total
*negative*	40	79	22	141
*neutral*	132	151	79	362
*positive*	296	283	205	784
total	468	513	306	1287

**Table 7:** The number of answers in Domain 4 (Figure 2, bottom left panel; p<0.0001; U=0.012321).

	Employees	Retirees1	Retirees2	Total
*A-1*	49	50	8	107
*A-2*	161	209	41	411
*A-3*	433	470	256	1159
*A-4*	457	544	380	1381
*A-5*	172	143	139	454
total	1272	1416	824	3512

**Table 8:** The number of answers in Domain 4 (Figure 2, bottom right panel; p<0.0001; U=0.012988).

	Employees	Retirees1	Retirees2	Total
*negative*	210	259	49	518
*neutral*	433	470	256	1159
*positive*	629	687	519	1835
total	1272	1416	824	3512

## Data Availability

The data supporting the findings of the article are available from the corresponding author upon request.
